# Novel Approach to Proficiency Testing Reveals Significant Variations in Biomarker Practice Leading to Critical Differences in Lung Cancer Management

**DOI:** 10.1016/j.jtocrr.2025.100837

**Published:** 2025-04-22

**Authors:** Kassandra R. Bisson, Andrea Beharry, Normand Blais, Michael D. Carter, Parneet K. Cheema, Patrice Desmeules, John G. Garratt, Barbara Melosky, Bryan Lo, Stephanie Snow, Basile Tessier-Cloutier, Edwin Tio, Stephen Yip, Jennifer R. Won, Brandon S. Sheffield

**Affiliations:** a Canadian Pathology Quality Assurance – Assurance qualite canadienne en pathologie (CPQA-AQCP), Richmond, British Columbia, Canada; b Division of Advanced Diagnostics, William Osler Health System, Brampton, Ontario, Canada; c Hematology-Oncology Service, Department of Medicine, Centre hospitalier de l’Universite de Montreal (CHUM), Montreal, Quebec, Canada; d Department of Pathology and Laboratory Medicine, Queen Elizabeth II Health Sciences Centre, Halifax, Nova Scotia, Canada; e Division of Medical Oncology, William Osler Health System, Brampton, Quebec, Canada; f Service of Anatomic Pathology and Cytology, Institut Universitaire de Cardiologie et de Pneumologie de Quebec, Universite Laval, Quebec City, Quebec, Canada; g Division of Medical Oncology, BC Cancer, Vancouver, British Columbia, Canada; h Division of Anatomical Pathology, The Ottawa Hospital, Ottawa, Ontario, Canada; i Division of Medical Oncology, Queen Elizabeth II Health Sciences Centre, Halifax, Nova Scotia, Canada; j Department of Pathology, McGill University Health Centre, Montreal, Quebec, Canada; k Department of Laboratory Medicine, William Osler Health System, Brampton, Ontario, Canada; l Department of Pathology and Laboratory Medicine, University of British Columbia, Vancouver, British Columbia, Canada

**Keywords:** Proficiency testing, Precision oncology, NSCLC, Biomarkers

## Abstract

**Introduction:**

Timely access to quality biomarker testing in NSCLC is critical to patient outcomes. The Canadian Pathology Quality Assurance provides external quality assurance (EQA) to laboratories in Canada. The Canadian Pathology Quality Assurance has recently developed a novel approach to molecular biomarker EQA testing, assessing accuracy, turnaround time, and interpretation of reports. This study reports the results of the first end-to-end biomarker EQA challenge in NSCLC.

**Methods:**

Three challenge specimens were made using NSCLC tissue and paired with clinical vignettes mimicking referred-in cases. Participants were to provide all required molecular testing (immunohistochemistry and gene sequencing) and submit final reports for each case, while being timed. Reports were assessed by molecular pathologists and medical oncologists who recommended a systemic treatment based on vignettes and reports.

**Results:**

A total of 13 Canadian laboratories participated. The turnaround time of molecular reporting ranged from five to 57 (median 22.5) calendar days. Two laboratories (15%) reported their results within 2 weeks. Four laboratories (31%) reported the results of their biomarkers after more than 30 days.

Only three laboratories received optimal status (23%). One laboratory (8%) failed due to a critical genotyping error, three (23%) received a suboptimal status due to inappropriately long turnaround times, and the remaining six (69%) received an adequate status.

**Conclusions:**

This report demonstrates the utility of this proficiency testing style compared with standard laboratory self-reporting. The approach has elucidated substantial differences in the quality of NSCLC biomarker results produced by Canadian laboratories. Ongoing efforts to improve turnaround times and clarity of reporting, including regular external measurement, are tools that can improve patient outcomes in NSCLC.

## Introduction

The treatment of NSCLC is heavily entwined with biomarker results.^1^ Biomarker-directed therapy is associated with superior outcomes for patients, compared with nontargeted chemotherapies, when appropriate.^2^

The presentation of NSCLC can be sudden, with death rates of untreated advanced NSCLC estimated at 4% per week.^3^ Outside of organized screening programs, most NSCLC cases present at advanced stage.^4^ Accessible biomarker results at the time of initiation of systemic therapy are known to be associated with significantly better patient survival.^5^

Laboratory-based care for patients with NSCLC is complex. Samples previously used for diagnostic histopathology or cytopathology are often referred to external institutions or separately managed molecular divisions for biomarkers that involve next-generation sequencing (NGS) and immunohistochemistry (IHC) for programmed death-ligand 1 (PD-L1) status, among others.^6^, ^7^, ^8^, ^9^

Laboratories typically engage in external quality assurance (EQA) to evaluate test accuracy. Many different schemes are available to laboratories internationally, testing various aspects of molecular pathology workflow.^10^, ^11^, ^12^, ^13^

In Canada, a publicly funded health care nation, many regions face poor access to biomarker testing.^14^ Lengthy turnaround times are often cited as a major barrier, where poor access to testing leads to non-biomarker–directed treatment decisions.^15^ Guidelines recommend that biomarkers be completed in a timely fashion (10 working days).^8^^,^^16^, ^17^, ^18^, ^19^ Complex, lengthy, and confusing molecular reports have also often been referenced by medical oncologists as prominent barriers to precision therapy.^20^, ^21^, ^22^

The Canadian Pathology Quality Assurance (CPQA) has been the leading EQA organization for IHC to Canadian laboratories.^23^ The CPQA recently described a novel approach to biomarker EQA.^24^ This end-to-end proficiency test attempts to measure the entire process of molecular testing to reporting, from accessioning to medical oncologist interpretation. Laboratory performance metrics include accuracy, reporting clarity, and turnaround time, among other preanalytic conditions believed to be the source of most biomarker-related morbidity.^25^

In this study, we report results of the first exercise comparing Canadian laboratories’ proficiency in delivering biomarker-directed therapy in NSCLC.

## Materials and Methods

Formalin-fixed paraffin-embedded (FFPE) blocks of surgically resected nonsquamous NSCLC were chosen from CPQA archives. Samples were then created using duplicate 2 mm punch of NSCLC tissue. All replicate samples originated from the same resected lung cancer (originally processed in 2022) and cases were chosen to accommodate the tissue requirements.

Three blocks, each with a unique clinical vignette, were distributed to each participating laboratory by tracked courier service. Each case was also submitted to a commercial Clinical Laboratory Improvement Amendments–certified laboratory for confirmatory reference genotyping.^26^ Challenge cases are summarized in Table 1.

**Table 1:** Summary of Challenge Cases 1 to 3

Case	Vignette	Genotype	PD-L1 Status
1	A 44-y-old man presenting to medical attention with diffusely metastatic disease to the bone, liver, and lymph nodes. He has no significant past medical history and has not used tobacco regularly. A supraclavicular lymph node biopsy result reveals adenocarcinoma, positive for TTF1, in keeping with metastatic NSCLC. Block from this biopsy has been referred to your laboratory for complete biomarker testing.	*EGFR* Exon 20 insertion c.2311delinsGGTT (p.Asn771delinsGlyTyr)	<1%
2	A 69-y-old man, currently on azathioprine for systemic lupus recently complicated by pericarditis. He has approximately 10 pack-year smoking history and quit 10 y ago. While being followed by his rheumatologist and cardiologist, he was found to have a new left lung mass with associated pleural effusion. Thoracentesis result reveals malignant cells. Biopsy of the lung mass reveals a poorly differentiated carcinoma, positive for keratin markers and negative for TTF1, p40 among others. Medical oncology is requesting full NSCLC biomarker testing on the lung biopsy. Please provide as soon as possible.	*MET* Exon 14 Skipping c.2942-14_2942-1del (p.?) *MET*(13)::*MET*(15) RNA fusion	<1%
3	A 73-y-old woman with a history of emphysema, severe COPD, and multiple additional medical comorbidities. She has a 40 pack-year tobacco history and continues to use tobacco daily. She was recently found to have a 7 cm left upper lobe lung mass with mediastinal lymphadenopathy; MRI brain confirms 3 foci of metastatic disease. Biopsy result of dominant LUL lesion reveals a TTF1-positive pulmonary adenocarcinoma. The biopsy sample has been referred to your laboratory for complete biomarker testing.	*KRAS* G12A c.35G>C (p.Gly12Ala)	1%–49%

COPD, chronic obstructive pulmonary disease; MRI, magnetic resonance imaging; PD-L1, programmed death-ligand 1.

Laboratory participants were asked to treat specimens as real referred-in cases, performing any necessary biomarker testing (which could include IHC and gene sequencing) that would be required for patient treatment and management. Final reports (IHC and molecular results) were to be submitted to the CPQA online portal. Participating laboratories were made aware that the exercise was being timed and that turnaround time would be measured as the date of receipt at the facility to the date that all three of the cases’ molecular biomarker reports were uploaded to the CPQA website.

An assessor team was assembled, consisting of medical oncologists and molecular pathologists. Assessors reviewed the clinical vignettes and reference results before the exercise and determined a criterion standard treatment for each case. Following the exercise, assessors were provided deidentified reports and then relayed commentary back to the CPQA for each report, including a systemic therapy prescription, through a structured online survey.

Analytical accuracy was assessed based on the concordance of genotype and PD-L1 status as compared with reference testing laboratory. PD-L1 status concordance was based on the ranges provided (i.e., <1%, 1%–49%, >50%) rather than on the raw tumor proportion score.

Total turnaround time was calculated in calendar days from the time the sample was delivered to the participant’s institution based on tracked shipping until the date of final report submission to the CPQA. Self-reported turnaround time is based on participant survey responses. Pearson correlation was calculated between the self-reported turnaround time and the measured total turnaround time or the time from accessioning to submission. Paired *t* tests with an alpha value of 0.05 were performed between (1) the self-reported and total turnaround times, (2) the self-reported and time from accessioning to submission, and (3) total turnaround time for integrated reports versus non-integrated reports. Bonferroni correction was also performed as needed. Statistical testing was done using R version 4.4.1.^27^

Report clarity was reviewed by the assessor committee based on the overall ease of identifying the pertinent information within the report, particularly if the results were recognizable to the medical oncologist assessors. Additional factors contributing to overall report clarity included whether the interpretations provided were correct and provided sufficient therapeutic or clinical context, if applicable.

Qualitative thematic analysis was also performed on the assessors’ laboratory-specific feedback acquired through the assessment survey open-text responses wherein significant phrases with similar meanings were extracted and categorized into common feedback themes.

Following the assessment of the reports, individual laboratory feedback was provided to all participants alongside a summary of the challenge results that could be used for interlaboratory comparison.

## Results

There were 17 laboratories known by the CPQA to provide NSCLC biomarker testing across Canada, and all were invited to participate in the exercise. A total of 13 laboratories agreed to participate and completed the exercise. Participating laboratories represented different provincial health care systems from across Canada, including British Columbia, Ontario, Quebec, prairie regions, and Atlantic Canada. One laboratory participant (laboratory 5) contacted CPQA following the exercise, indicating that their measured turnaround time was felt to be inaccurate as their hospital-based electronic medical record was unable to access the fictitious test cases in alignment with the exercise. This participant (laboratory 5) was not included in turnaround time statistics. Three laboratories (3, 5, 10) were excluded in the statistical testing calculations for self-reported turnaround time comparison due to incomplete survey data.

**Figure 1:**
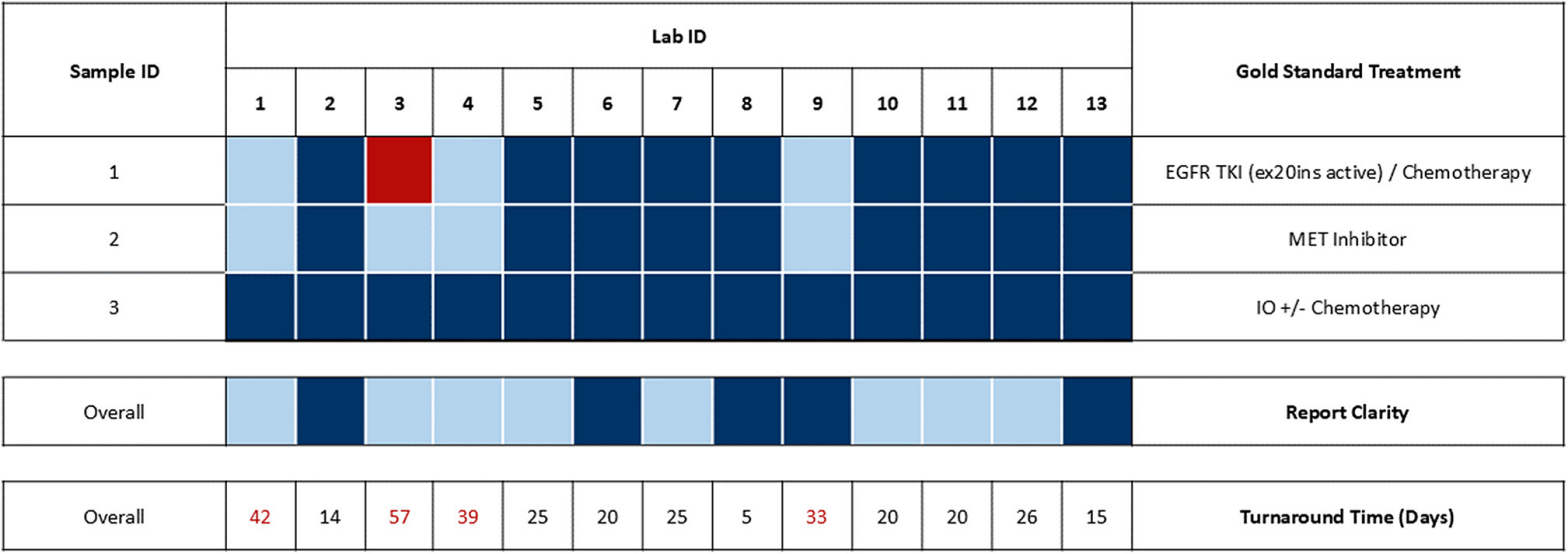
Assessment summary for all laboratories. Gold standard treatment: Dark blue depicts concordance between assessor responses for correct treatment recommendation. Light blue depicts heterogeneity among assessors’ treatment recommendation. Red denotes a unanimously incorrect treatment recommendation. Report clarity: Dark blue depicts assessor concordance of adequate report clarity. Light blue depicts heterogeneity among assessor evaluation of report clarity. Turnaround time: Calculated in calendar days. Red font depicts turnaround times deemed suboptimal by assessors.

**Figure 2:**
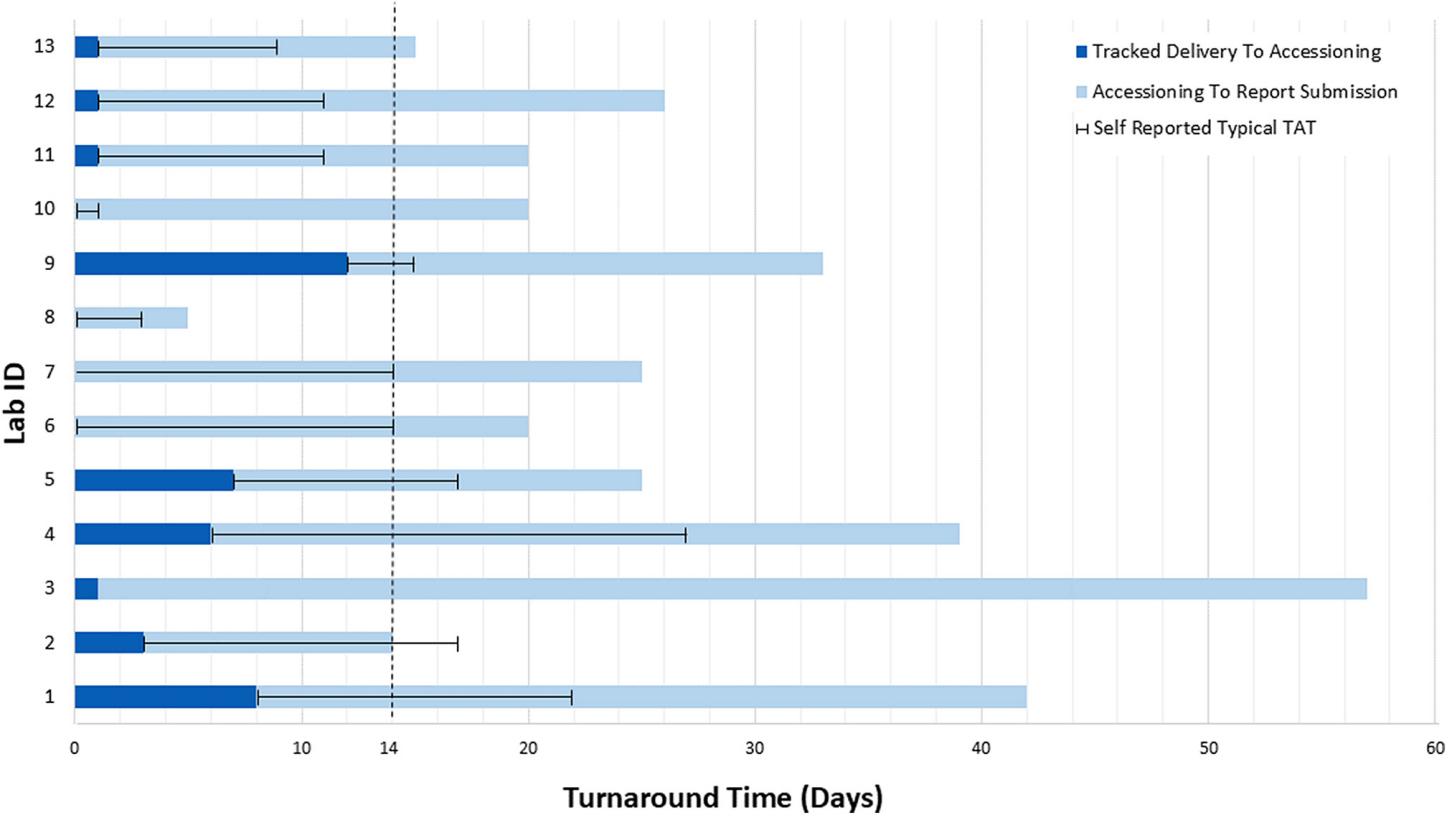
Challenge TATs in calendar days by laboratory. “0” day marks the date of specimen delivery at the participating institution as reported by tracked commercial courier. Laboratory IDs 1 to 13 (901–913). Dark blue bars represent time from delivery of specimen to accessioning date. Light blue bars depicting time from accessioning to final online report submission. Black lines depicting self-reported typical TAT from date of accessioning. Dashed line identifying the maximum TAT of 14 calendar days from receipt of blocks, as per Canadian testing guidelines. ∗Laboratory 5 not included in the statistical calculations for TAT (see Results section for details). TAT, turnaround time.

**Figure 3:**
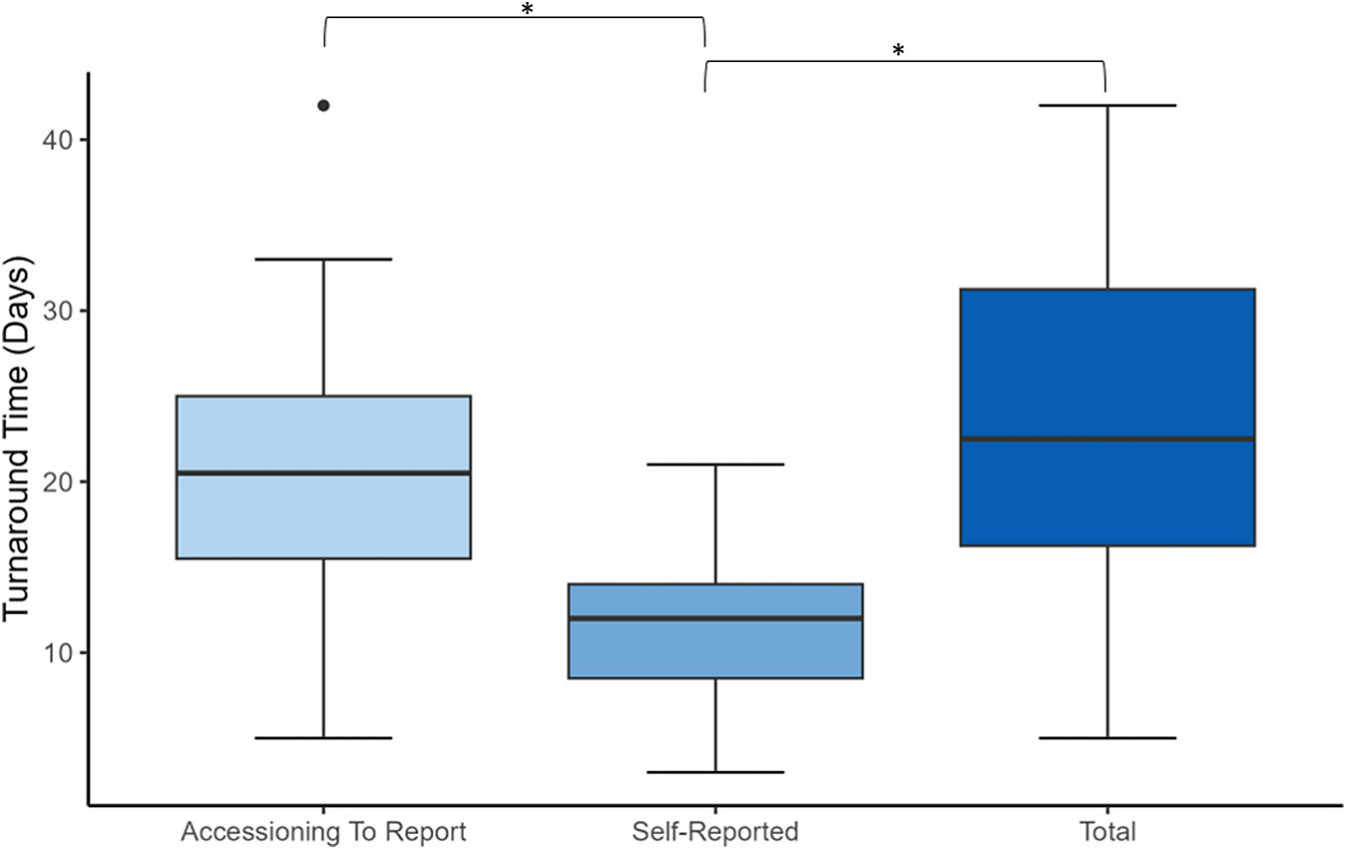
Boxplot of challenge TATs in calendar days for laboratory participants. ∗ Denotes significant difference (*p* value < 0.05) between laboratory self-reported typical TAT and TAT from receipt of specimen to final report submission (Total) or from date of accessioning to final report submission (Accessioning To Report). TAT, turnaround time.

Total turnaround time ranged between 5 and 57 (median 22.5, interquartile range 18.75–34.5) calendar days. Two of 13 (15%) laboratories were able to submit full biomarker reports within 2 weeks. Four laboratories (31%) took over 30 days to report biomarkers. Every participant’s measured turnaround time was greater than their self-reported turnaround time (range 3–21 days), with the exception of laboratory 2, which met their self-reported turnaround time. There was only moderate correlation between the laboratories’ self-reported turnaround times and their measured turnaround time (correlation coefficient = 0.475). There was stronger correlation between the self-reported turnaround time and the turnaround time from accessioning to submission (correlation coefficient = 0.58). The self-reported turnaround times were statistically different from the accessioning turnaround time (*p* < 0.05) and the total turnaround time (*p* < 0.05). Time between sample delivery and accessioning ranged between 0 and 12 days (median 1 day). Turnaround time data are found in Figure 1, Figure 2, Figure 3.

Overall accuracy in biomarker results was excellent, with 97% concordance to reference laboratory for genotyping results and a 100% concordance rate for PD-L1 assessment. A single, yet major, genotyping error was observed, where one laboratory failed to identify an *EGFR* exon 20 insertion in case 1.

Additional minor discrepancies, not affecting laboratory’s performance status, included the following: inability to detect the *MET* exon 14 skipping DNA variant in case 2 (identification only of *MET*(13)::*MET*(15) skipping reads in tumor RNA) and slight base calling differences in insertion/deletions in *EGFR* and *MET* for cases 1 and 2. Two laboratories failed to sequence the RNA for cases 1 and 2, citing the quality or quantity of the tissue provided as the limiting factor despite the remaining laboratories able to sequence RNA on the same case.

Various sequencing methodologies were used by the participants and are summarized in Table 2. All laboratories except for one used the 22C3 PD-L1 clone, with the remaining laboratory using the SP263 clone.

**Table 2:** NGS Platforms Used by Challenge Laboratory Participants

Laboratory ID	Assay	Platform
1	AmpliSeq Focus Panel	Illumina
2	AmpliSeq Focus Panel	Illumina
3	Oncomine Comprehensive Assay v3 (Oncomine Comprehensive Plus for RNA)	Thermo Fisher
4	Oncomine Precision Assay	Thermo Fisher
5	Oncomine Comprehensive Assay v3	N/A
6	Oncomine Comprehensive Assay v3 (Oncomine Comprehensive Assay Plus for RNA)	Thermo Fisher
7	Oncomine Comprehensive Assay v3	N/A
8	Oncomine Precision Assay	Thermo Fisher
9	AmpliSeq Focus	N/A
10	Oncomine Comprehensive Assay Plus	Thermo Fisher
11	AmpliSeq Focus for Illumina	Illumina
12	Solid Tumor Panel—not otherwise specified	Thermo Fisher
13	Archer Analysis v7 (DNA), Archer Fusion Plex Lung v2 (RNA)	Illumina

*Note:* N/A indicates information not provided or ascertainable through reports.

ID, identification; NGS, next-generation sequencing.

A variety of reporting styles was observed with reports from five laboratories deemed consistently clear and easy to understand by medical oncologist assessors (Fig. 1). These laboratories were found to have integrated IHC and molecular result summaries in their report and used colloquial terminology (i.e., “*EGFR* exon 20 insertion”) beyond the more formal p. or c. HGVS nomenclature (i.e., *EGFR* (NM_005228.5):c.2311delinsGGTT, p.(Asn771delinsGlyTyr)).

**Figure 4:**
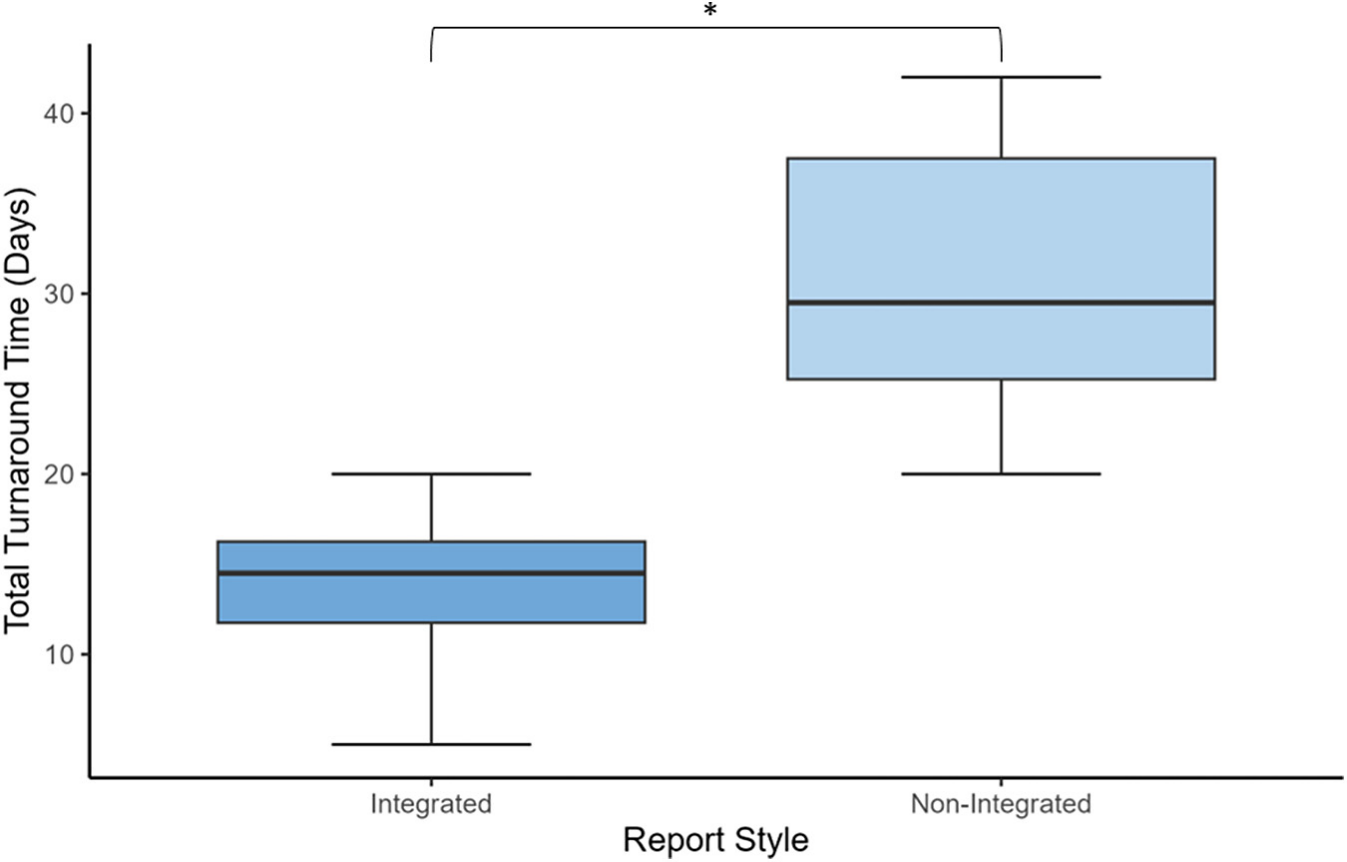
Boxplot of challenge turnaround times in calendar days for laboratory participants using integrated reports versus non-integrated reports. ∗Denotes significant difference (*p* value < 0.05) between turnaround times of laboratory participants that used an integrated reporting style and those that did not.

The reports ranged between 1 and 27 pages (median 3). Four laboratories (31%) had separate reports for their molecular and IHC results. Nine of 13 laboratories had a single report that contained both NGS and IHC biomarker results. Of those nine, four had integrated reports, where immunohistochemical biomarkers (PD-L1) could be found in conjunction with genotypic (NGS) information in a single document, released at a single time, by a single signatory. The turnaround time was found to be significantly different between the groups that used integrated reports and those that did not (*p* < 0.05) (Fig. 4).

Nine of 13 laboratories’ reports (69%) led medical oncologist assessors to the gold standard treatment in all three cases. Despite accurate genotyping, four laboratories were unable to guide medical oncologist assessors to the gold standard treatment due to protracted turnaround time (>30 d). Three of 13 laboratories (23%) received an optimal status by providing timely, accurate, and easily interpretable biomarker reports leading medical oncologist assessors to gold standard treatment. Summary of the assessment is depicted in Figure 1. One laboratory (8%) failed due to a critical genotyping error leading to mistreatment in case one, three additional laboratories (23%) received a suboptimal status due to inappropriately long turnaround times, and the remaining (46%) received an adequate status.

From the assessor survey data, the following four major themes were identified:

1. Delays (long turnaround times detract from accuracy of results): All assessors commented that turnaround times more than 30 days were not received with enough time to deliver biomarker-informed treatment without interrupting patient flow. For example, “57 day turnaround time is not clinically helpful and in fact harmful, the laboratory should refer to another laboratory for this testing. This patient would have been dead before the biomarkers were back if we didn’t treat him.” assessor 7, case 1
2. Report length and organization (difficulty locating pertinent results across many pages): All assessors commented about concerns with length and organizations of at least one report. For example, “You have to read through so much of the actual methodology of testing to get to the result you need. The result was in the same font and size as all the non-relevant text. […] This is a piecemeal report and the oncologist is left to comb through the report to find the important information.” assessor 7, case 1
3. Terminology (use of colloquial terms in results improves clinician understanding): Five of seven assessors noted instances of noncolloquial terminology being difficult to identify or understand. For example, “they didn’t actually say *KRAS* G12A anywhere – just written in the format with amino acid names, lots of stuff to wade through before you get to the actual mutation.” assessor 6, case 3
4. Interpretation (vague or extensively long interpretations detract from accurate results): All assessors made note of result interpretation sections that had key information about the clinical significance of the results either missing or buried. For example, “Thorough but extremely long report and too many text & annotations - at least add page numbers. Extensive description of individual gene information not clinically relevant but potentially clinically relevant information such as low coverage regions is missing.” assessor 4, case 1

## Discussion

Optimal outcomes in NSCLC treatment are heavily predicated on biomarker access. High-quality biomarker results are required to not only be accurate, but timely, and comprehendible by end users. This study is among the first to explore a nearly complete end-to-end process from receipt of an FFPE sample to the prescription of a systemic therapy by a medical oncologist. The results presented here highlight and quantify many of the struggles that arise in the treatment of NSCLC.

### Analytic Accuracy

Traditional quality assurance programs focus primarily on the analytic accuracy of results.^11^^,^^12^ These are typically performed for IHC and genetic biomarkers.^11^, ^12^, ^13^ In this exercise, a very high accuracy rate was observed for both PD-L1 and genetic results. A single major genotyping error was observed where one laboratory failed to identify and report an *EGFR* exon 20 insertion driver mutation. This would have resulted in a significant change in treatment should the error have occurred in a real-world setting. Similar to traditional EQA models, a notification of the failure was provided to the participating laboratory and the information gained in the exercise should be used to identify and correct the source of the error.

Traditional molecular EQA programs provide DNA as a substrate. This would require laboratories to test only one component of their biomarker pipeline (mainly gene sequencing or other genotyping methods). Accurate results are well known to be a culmination of many interrelated processes, often performed by different individuals in various parts of the laboratory. By using FFPE blocks, accurate results would necessarily require high performance in tissue assessment, microtomy, DNA (or RNA) extraction, sequencing, and bioinformatic analysis. Errors in any of these processes can result in suboptimal patient care.

The management of NSCLC has grown increasingly complex, requiring close multidisciplinary collaboration, with the frequent utilization of multidisciplinary tumor boards, with biomarker data being relevant at every stage of treatment. This shift is necessitating a more complex approach to quality assurance in biomarker testing.

### Turnaround Time

Turnaround time is a critical factor in delivering high-quality biomarker results, especially in NSCLC due to the aggressive nature of the disease, but has seldom or never been objectively measured as has been done in this exercise.^28^ Although EQAs provide due dates for results submission, these typically provide several weeks for completion and are not intended to measure turnaround time. Many quality-related surveys collect self-reported laboratory turnaround time data; however, there is limited external monitoring or evaluation of this. The results of this study reveal that self-reported turnaround time data are not accurate. The data suggest that using self-reported turnaround time can mask underlying quality issues.

Many laboratories will define and measure turnaround time “from receipt in the laboratory” or from the date of accessioning. The data collected here reveal a wide variability in how long specimens waited to be accessioned after receipt at a testing facility, ranging from 0 to 12 days. This component of turnaround time represents an obvious area for improvement for many laboratories. Although often neglected, this element of turnaround time may be easily remedied, as many participants were able to have accession specimens on the day of delivery. One limitation of this study is that it only explores turnaround time components within a testing center. Institutions referring out for biomarker results also play a key role in turnaround time and should make every effort to ensure specimens are received at a testing facility within three days of a NSCLC diagnosis.^18^ To achieve this, referring institutions should practice reflex testing, avoid batch shipping, and use fast and reliable courier services, among other measures.^29^

In terms of total turnaround time, four laboratories (31%) were found to have unacceptably high turnaround times of more than 30 days (double the upper limit dictated in Canadian and international guidelines).^8^^,^^16^, ^17^, ^18^^,^^30^ One laboratory took 57 days to report results, and this unfortunately does reflect the clinical experience of many practicing oncologists in Canada.^14^^,^^15^ All reviewers found these four turnaround times to be unacceptable. As the participating institutions who produced these protracted turnaround times are all in good standing with traditional EQA programs and regional regulatory bodies, the results presented here highlight the need for an improved approach to biomarker EQA and laboratory regulation. Many assessors commented that long turnaround times detracted from the accuracy of the results and would be harmful to patients awaiting treatment, emphasizing the importance of external turnaround time monitoring through EQAs.

Three laboratories had optimal results with turnaround times of 15 days or less. Within Canada, a publicly funded health care nation, all participating laboratories would have similar resources to work with, and the fact that three laboratories could produce such streamlined results is encouraging. Each of the three laboratories with optimal turnaround time achieved this with different gene sequencers and different NGS panels (Table 2). All these laboratories were noted to use integrated reports, with a single pathologist reporting both NGS and IHC biomarkers. This is believed to be a critical component in high-quality biomarker delivery and was associated with significantly improved turnaround time. Conversely, fractionated reports with independent NGS and PD-L1 interpretation were not only slower, but more difficult for oncologists to interpret. The fractionation displayed within the reports may be indicative of a more convoluted specimen journey, requiring multiple laboratory sections or facilities, leading to delayed turnaround time.

All laboratories were instructed at multiple time points before the exercise that turnaround time would be measured and that participants should treat the test samples as they would a clinical sample. One laboratory (laboratory 5) contacted the CPQA following the exercise indicating that the 25-day turnaround time measured was not reflective of clinical practice due to difficulties accessioning the sham patient specimens. In all other cases, the measured turnaround times are presumed to be reflective of clinical practice and felt to reflect the experience of Canadian medical oncology practitioners.

### Report Clarity

In addition to accuracy and timeliness, results must be understood and correctly interpreted by oncologists. Reviewers noted several factors that led to suboptimal interpretation of reports.

One of the prominent themes from assessor feedback was around the terminology and nomenclature used within the reports. The isolated use of HGVS nomenclature to communicate results was viewed as highly problematic. Although some guidelines have emphasized the importance of HGVS nomenclature, oncologists are not able to easily recognize or interpret these very long alphanumeric symbols for mutations.^20^^,^^28^^,^^31^, ^32^, ^33^, ^34^ As a result, there has been a shift in more recent guidelines and practices toward emphasizing the use of “colloquial nomenclature” to ensure that end users can easily identify the genotypes for clinical decision-making.^20^^,^^22^^,^^32^ This would entail the use of single-letter amino acid abbreviations, that is, *"KRAS* G12D” instead of “*KRAS* (NM_033360.4):c.35G>A, p.(Gly12Asp).” Furthermore, for more complex variants, optimal reporting using colloquial nomenclature would include descriptive terminology such as “*MET* exon 14 skipping” or “*EGFR* exon 20 insertion” as opposed to “*MET* (NM_001127500.3):c.2942-14_2942-1del(p.?)” or “ENST00000397752(MET):e.13::ENST000000397752(MET):e.15” and *EGFR* (NM_005228.5):c.2311delinsGGTT;p.N771delinsGY.” Although full, formal HGVS nomenclature could be included on a report, these data prompt a stern warning to report-writers that this nomenclature is not well understood or recognized by medical oncologists.

Additional prevalent themes from the assessor feedback highlighted concerns about the length of these reports in terms of number of pages but also in length of interpretation content. Many of the reports were found to be lengthy and complicated. Lengthy descriptions regarding the biology of well-known driver genes and their common mutations were found to be unnecessary and distracted end-users from the most pertinent details. Optimal annotations were focused directly on the clinical implications of the findings with respect to patient treatment, in the context of the clinical vignettes. Additional details on protein biology, prognostic information, and other details were all found to detract from overall report clarity, particularly when there were results of far more critical importance. Generally, optimal report clarity could be achieved in a report of 2 pages.

As stated previously, optimal results were achieved with a single report containing both NGS and IHC biomarkers. In cases where a single pathologist reporter of both the IHC and NGS results is not possible due to the organization of the molecular and IHC laboratories and workflows, a summary statement or section near the top of the report with both pertinent NGS and IHC data was found to greatly assist in report interpretation.

### Systemic Therapy

The biomarker testing process is complex and multifactorial, including the final interpretation of a report by end-users with varying degrees of knowledge and experience. This study is the first of its kind to use systemic therapy decision as an end point for laboratory EQA in lung cancer. This end point is dependent on innumerable variables, all culminating with a final clinical decision.

The approach offers considerable advantages in the face of a complex measurement. For instance, laboratory 3 failed to identify a key driver mutation, leading all oncologists to inappropriately prescribe this patient an immunotherapy-containing regimen. Laboratory 4 did correctly identify the driver mutation for this patient; however, it was not reported for 39 days. Even though the result was analytically accurate, the turnaround time was inappropriate and led to a heterogeneous set of treatment decisions by oncologist reviewers. Despite the fact that the errors committed by laboratories 3 and 4 were different, they both led oncologists away from the most appropriate treatment in case 1.

As lung cancer treatment has grown highly complex and multidisciplinary in nature, quality assessment and monitoring must keep pace accordingly. The use of systemic therapy prescription as a quality end point and including clinical oncologists in the EQA process represents a unique approach to laboratory quality management and should be considered for more regular and widespread adoption.

In conclusion, state-of-the-art treatment in lung cancer is entirely predicated on biomarker results. To achieve optimal outcomes, biomarkers must be accurate and available in a clinically relevant time frame.

In this study, we report for the first time, the utility of end-to-end biomarker testing in NSCLC. This approach can clearly delineate the performance of laboratories with respect to supporting precision oncology practice. This approach can be used to achieve equity in lung cancer treatment by exposing regions and practices needing improvement. The approach can identify high-performing laboratories capable of defining and transferring best practice styles. The results of this study suggest that wider and more frequent use of end-to-end proficiency testing could be a valuable tool in improving outcomes for patients with lung cancer.

Key recommendations from the results of this study to improve the quality of precision oncology practice include the following:

1. Minimize transport and transmission delays in biomarker reporting.
2. Focus report annotations to be interpretable by medical oncologists.
3. Laboratories should regularly participate in proficiency testing and quality improvement initiatives.

This study showcases the utility of end-to-end style of proficiency testing in NSCLC within Canada. This style of quality improvement exercise may provide utility in improving biomarker access and wider accessibility to precision cancer therapy.

## CRediT Authorship Contribution Statement

**Kassandra R. Bisson:** Conceptualization, Methodology, Formal analysis, Data curation, Visualization, Investigation, Writing – Original Draft, Writing – Review & Editing.

**Andrea Beharry:** Resources, Writing – Review & Editing.

**Normand Blais:** Investigation, Writing – Review & Editing.

**Michael D. Carter:** Investigation, Writing – Review & Editing.

**Parneet K. Cheema:** Resources, Writing – Review & Editing.

**Patrice Desmeules:** Resources, Writing – Review & Editing.

**John G. Garratt:** Writing – Review & Editing.

**Barbara Melosky:** Resources, Writing – Review & Editing.

**Bryan Lo:** Writing – Review & Editing.

**Stephanie Snow:** Resources, Writing – Review & Editing.

**Basile Tessier-Cloutier:** Resources, Writing – Review & Editing.

**Edwin Tio:** Resources, Writing – Review & Editing.

**Stephen Yip:** Resources, Writing – Review & Editing.

**Jennifer R. Won:** Project administration, Methodology, Data curation, Investigation, Writing – Review & Editing.

**Brandon S. Sheffield:** Conceptualization, Methodology, Investigation, Writing – Original Draft, Writing – Review & Editing.

## Disclosure

There are not perceived to be any conflicts of interest affecting the integrity of the data presented here. Individual author financial disclosures: Dr. Carter is a member of advisory board and has received honoraria from Amgen, AstraZeneca, Bayer, InCyte, Janssen, Merck, Novartis, and Pfizer; has received research grant from 10.13039/100004325AstraZeneca. Dr. Cheema is an advisory board member of Amgen, AstraZeneca, Bristol Myers Squibb, Roche, Boehringer Ingelheim, Bayer, Roche, Novartis, Merck, oncologyeducation, and Pfizer; has received honorarium from Thermo Fisher, Merck, AstraZeneca, Roche, and oncology education; and has received research grants from AstraZeneca. Dr. Desmeules is a member of the advisory board/provided consulting for Roche, AstraZeneca, and Merck; has received research support from AstraZeneca, 10.13039/100004319Pfizer, 10.13039/100004336Novartis, 10.13039/100004337Roche, 10.13039/100004755EMD Serono, Eli Lilly, Amgen, and Merck. Dr. Melosky is a member of the advisory board/has received honorarium from Amgen, AstraZeneca, BI, Bristol Myers Squibb, EMD Serono, GlaxoSmithKline, Janssen, Merck, Novartis, Pfizer, Roche, and Takeda. Dr. Lo is an advisory board member of AstraZeneca, Pfizer, Bayer, Novartis, Jansen, and Roche; medical director for the Ottawa Molecular Oncology Diagnostics Laboratory which has received research and quality improvement grant support from 10.13039/100002429Amgen, AstraZeneca, Roche, and EMD Serono. Dr. Snow is a member of the advisory boards/provided consulting for Amgen, Bayer, Beigene, Boehringer Ingelheim, Astellas, AstraZeneca, Bristol Myers Squibb, Daiichi Sankyo, Janssen, Knight, Lilly, Merck, Merck Sharp & Dohme, Novartis, Pfizer, Roche, Sanofi, Taiho, and Takeda; has research trials (institutional funding) for Amgen, ARCUS, AstraZeneca, Bristol Myers Squibb, GlaxoSmithKline, Merck, Novartis, and Sanofi; and board of directors and president of Lung Cancer Canada. Dr. Yip is a member of advisory board and has received honoraria from Amgen, AstraZeneca, Bayer, Pfizer, Roche, and Servier. Dr. Sheffield has received honoraria, grant support, and/or participated in advisory meetings with Amgen, AstraZeneca, Bayer, Biocartis, Boehringer Ingelheim, Cell Marque, Elevation Oncology, Eli Lilly, EMD Serono, Incyte, Janssen, Merck, Novartis, Pfizer, Roche, Sanofi, Thermo Fisher, and Turning Point Therapeutics. The remaining authors declare no relevant conflicts of interest.

## References

[1] Ionescu D.N., Stockley T.L., Banerji S. (2022). Consensus recommendations to optimize testing for new targetable alterations in non-small cell lung cancer. Curr Oncol.

[2] Kris M.G., Johnson B.E., Berry L.D. (2014). Using multiplexed assays of oncogenic drivers in lung cancers to select targeted drugs. JAMA.

[3] Stewart D.J., Maziak D.E., Moore S.M. (2021). The need for speed in advanced non-small cell lung cancer: a population kinetics assessment. Cancer Med.

[4] Chen V.W., Ruiz B.A., Hsieh M.-C., Wu X.-C., Ries L.A.G., Lewis D.R. (2014). Analysis of stage and clinical/prognostic factors for lung cancer from SEER registries: AJCC staging and collaborative stage data collection system. Cancer.

[5] Aggarwal C., Marmarelis M.E., Hwang W.T. (2023). Association between availability of molecular genotyping results and overall survival in patients with advanced nonsquamous non-small-cell lung cancer. JCO Precis Oncol.

[6] Reisman D.N., Sciarrotta J., Wang W., Funkhouser W.K., Weissman B.E. (2003). Loss of BRG1/BRM in human lung cancer cell lines and primary lung cancers: correlation with poor prognosis. Cancer Res.

[7] Agaimy A., Fuchs F., Moskalev E.A., Sirbu H., Hartmann A., Haller F. (2017). SMARCA4-deficient pulmonary adenocarcinoma: clinicopathological, immunohistochemical, and molecular characteristics of a novel aggressive neoplasm with a consistent TTF1neg/CK7pos/HepPar-1pos immunophenotype. Virchows Arch.

[8] Lindeman N.I., Cagle P.T., Aisner D.L. (2018). Updated molecular testing guideline for the selection of lung cancer patients for treatment with targeted tyrosine kinase inhibitors: guideline from the College of American Pathologists, the International Association for the Study of Lung Cancer, and the Association for Molecular Pathology. J Mol Diagn.

[9] Brahmer J.R., Tykodi S.S., Chow L.Q.M. (2012). Safety and activity of anti-PD-L1 antibody in patients with advanced cancer. N Engl J Med.

[10] Müller C.R. (2001). European Molecular Genetics Quality Network. Quality control in mutation analysis: the European molecular genetics quality network (EMQN). Eur J Pediatr.

[11] EMQN. Our EQAS. https://www.emqn.org/our-eqa-schemes/.

[12] College of American Pathologists Proficiency testing. https://www.cap.org/laboratory-improvement/proficiency-testing.

[13] Laudus N., Nijs L., Nauwelaers I., Dequeker E.M.C. (2022). The significance of external quality assessment schemes for molecular testing in clinical laboratories. Cancers.

[14] Snow S., Brezden-Masley C., Carter M.D. (2024). Barriers and unequal access to timely molecular testing results: addressing the inequities in cancer care delays across Canada. Curr Oncol.

[15] Fleming K.E., Hupel A., Mithoowani H., Lulic-Kuryllo T., Valdes M. (2024). Biomarker turnaround times and impact on treatment decisions in patients with advanced non-small cell lung carcinoma at a Large Canadian community hospital with an affiliated Regional Cancer Centre. Curr Oncol.

[16] Cheema P.K., Banerji S.O., Blais N. (2023). Canadian consensus recommendations on the management of KRAS G12C-mutated NSCLC. Curr Oncol.

[17] Cheema P.K., Banerji S.O., Blais N. (2021). Canadian consensus recommendations on the management of MET-altered NSCLC. Curr Oncol.

[18] Cheema P.K., Gomes M., Banerji S. (2020). Consensus recommendations for optimizing biomarker testing to identify and treat advanced EGFR-mutated non-small-cell lung cancer. Curr Oncol.

[19] Lim C., Sekhon H.S., Cutz J.C. (2017). Improving molecular testing and personalized medicine in non-small-cell lung cancer in Ontario. Curr Oncol.

[20] West H.J., Lovly C.M. (2023). Ferrying oncologists across the chasm of interpreting biomarker testing reports: systematic support needed to improve care and decrease disparities. JCO Oncol Pract.

[21] Schmid S., Jochum W., Padberg B. (2022). How to read a next-generation sequencing report-what oncologists need to know. ESMO Open.

[22] Malapelle U., Leighl N., Addeo A. (2024). Recommendations for reporting tissue and circulating tumour (Ct)DNA next-generation sequencing results in non-small cell lung cancer. Br J Cancer.

[23] Sheffield B.S., Garratt J., Kalloger S.E. (2014). HER2/Neu testing in gastric cancer by immunohistochemistry: assessment of interlaboratory variation. Arch Pathol Lab Med.

[24] Bisson K.R., Won J.R., Beharry A. (2024). Novel approach to proficiency testing highlights key practice variations in cancer biomarker delivery. J Mol Pathol.

[25] Compton C.C., Robb J.A., Anderson M.W. (2019). Preanalytics and precision pathology: pathology practices to ensure molecular integrity of cancer patient biospecimens for precision medicine. Arch Pathol Lab Med.

[26] Prentice L.M., Miller R.R., Knaggs J. (2018). Formalin fixation increases deamination mutation signature but should not lead to false positive mutations in clinical practice. PLoS One.

[27] R Core Team R: a language and environment for statistical computing. https://www.R-project.org/.

[28] Dufraing K., Fenizia F., Torlakovic E. (2021). Biomarker testing in oncology – requirements for organizing external quality assessment programs to improve the performance of laboratory testing: revision of an expert opinion paper on behalf of IQNPath ABSL. Virchows Arch.

[29] Gosney J.R., Paz-Ares L., Jänne P. (2023). Pathologist-initiated reflex testing for biomarkers in non-small-cell lung cancer: expert consensus on the rationale and considerations for implementation. ESMO Open.

[30] Lindeman N.I., Cagle P.T., Beasley M.B. (2013). Molecular testing guideline for selection of lung cancer patients for EGFR and ALK tyrosine kinase inhibitors: guideline from the College of American Pathologists, International Association for the Study of Lung Cancer, and association for molecular pathology. Arch Pathol Lab Med.

[31] Cree I.A., Deans Z., Ligtenberg M.J.L. (2014). Guidance for laboratories performing molecular pathology for cancer patients. J Clin Pathol.

[32] Li M.M., Datto M., Duncavage E.J. (2017). Standards and guidelines for the interpretation and reporting of sequence variants in cancer: a joint consensus recommendation of the association for molecular pathology, American Society of Clinical Oncology, and College of American Pathologists. J Mol Diagn.

[33] Deans Z.C., Ahn J.W., Carreira I.M. (2022). Recommendations for reporting results of diagnostic genomic testing. Eur J Hum Genet.

[34] Richards S., Aziz N., Bale S. (2015). Standards and guidelines for the interpretation of sequence variants: a joint consensus recommendation of the American College of Medical Genetics and Genomics and the association for molecular pathology. Genet Med.

